# Socio-Demographic Characteristics and Sexual Behavioral Factors of Patients with Sexually Transmitted Infections Attending a Hospital in Southern Italy

**DOI:** 10.3390/ijerph18094722

**Published:** 2021-04-28

**Authors:** Teresa Fasciana, Giuseppina Capra, Paola Di Carlo, Cinzia Calà, Marco Vella, Giuseppe Pistone, Claudia Colomba, Anna Giammanco

**Affiliations:** 1 Department of Health Promotion, Mother and Child Care, Internal Medicine and Medical Specialties, University of Palermo, 90127 Palermo, Italy; giuseppina.capra@unipa.it (G.C.); paola.dicarlo@unipa.it (P.D.C.); cinzia.cala@unipa.it (C.C.); giuseppe.pistone@unipa.it (G.P.); claudia.colomba@unipa.it (C.C.); anna.giammanco@unipa.it (A.G.); 2 Department of Surgical, Oncological and Stomatological Sciences, Section of Urology, University of Palermo, 90127 Palermo, Italy; marco.vella@unipa.it

**Keywords:** STIs, behaviour risky factors, MSM, HPV, syphilis

## Abstract

Sexually transmitted infections (STIs) are a serious global health problem. In Italy, data describing the vulnerability to STIs of specific sexual minorities and the influence of sociodemographic and behavioral determinants are limited, as most infections are not subject to mandatory notification. This retrospective study describes the sociodemographic profile and main sexual behaviors of patients attending a hospital in Palermo (Sicily, Italy) from January 2018 to March 2019 as predictors of STI risk. Patients were divided in subgroups: men-who-have-sex-with-men (MSM), men-who-have-sex-with-women (MSW), bisexual men and females. Data were obtained through an anonymous questionnaire. Patients were tested for chlamydia, syphilis, *Mycoplasma genitalium* infection, genital herpes and HPV infection. A total of 294 subjects with STIs (male/female ratio about 2:1) were screened. Of the total sample, 79.6% of patients were Italian. MSM accounted for 34.3%, MSW for 29.6%, bisexual men for 5.8% and females for 30.3%. A total of 44.5% of patients had a high education level, 42.5% reported irregular use of condoms, 20.7% reported having had 5–10 partners in the six months prior to the visit and 32.9% were HIV-positive. HPV infection and syphilis were the most prevalent STIs. Conclusions: The most common profile of patients attending our clinic was that of an adult, Italian man with a high level of education, poor use of condoms and a high number of partners. MSM had the highest sex-behavior-related risk for STIs. In addition, our results suggest that all STD teams need to implement counselling topics and recommendations to share with patients, as well as tips on how to approach sexual health education/counselling, thereby promoting patient-centered approaches and educational programs.

## 1. Introduction

Sexually transmitted infections (STIs) are among the most important global public health problems both in high-income and low-income countries [1]. STI increase worldwide is mostly due to changes in sexual behaviors, such as contact with a significant number of sexual partners, the lower average age of people engaging in sexual intercourse and inconsistent condom use [2].

In 2016, the World Health Organization (WHO) estimated 376 million new cases (more than 1 million per day) of curable STIs (i.e., chlamydia, gonorrhea, syphilis and trichomoniasis). The burden of viral STIs is similar, with an estimated 417 million prevalent cases of herpes simplex virus infection (HSV) and about 291 million women infected with human papillomavirus (HPV) [1]. Regarding the STI scenario in Italy, this is mainly defined by the Istituto Superiore di Sanità (ISS) that periodically updates the surveillance system based on data received from clinical centers and laboratories located throughout the country [3,4]. Despite this, few data are available about the epidemiology of STIs in Italy because most infections are not subject to mandatory notification [4]. According to ISS, the most frequently registered STIs in Italy from 1991 to 2017 were anogenital HPV infection (42.4%), latent syphilis (8.4%), genital herpes (7.3%) and non-chlamydial non-gonococcal bacterial cervicovaginitis (7.1%) [4]. Regarding asymptomatic STIs cases, previous Italian studies reported high incidence in the female general population as well as in the HIV subgroup [5,6,7].

The emergence of HIV has highly influenced the epidemiology and clinical pattern of STIs. In Europe, the number of reported STIs decreased due to the fear of HIV infection. However, since the beginning of this century when the status of HIV infection changed to a chronic disease, a steady increase has been re-recorded [8,9]. Correlations between HIV and STIs are multiple: ulcerative STIs (chancroid, syphilis and herpes) facilitate the sexual transmission of HIV by increasing both infectivity and susceptibility and can increase the risk of HIV acquisition (threefold or more). Furthermore, the co-presence of HIV may trigger the progression of infectious diseases, and STIs may be more difficult to treat while the symptomatic phases may be lengthened. Emphasis on the relationship between STIs and HIV has led to attempts to reduce HIV incidence through increased STI testing and treatment; for this reason, it is important to rethink research goals and prophylaxis interventions in STIs [10,11].

Moreover, STIs and HIV share the same behavioral, socio-economic and demographic risk factors, such as age at first sexual intercourse, low condom use, multiple sexual partners, being single and the sexual behavior of partners (different sexual behavior with main vs. other partners). In this regard, studies revealed the existence of population groups particularly vulnerable to STIs, such as immigrants, adolescents, sex workers, men who have sex with men (MSM) and bisexuals [12,13,14,15,16,17]. MSM are a sexual minority of particular interest because of the increase in the prevalence of HIV and other STIs in recent decades [17]. Individual risks of HIV acquisition in MSM have been associated with condomless anal receptive sex, high frequency of male partners, injection drug use (IDU), high viral load in index partner and amphetamine-type stimulants [15].

Based on these considerations, a sample of patients with a confirmed diagnosis of STI attending an STI Unit of the Policlinico “Paolo Giaccone” in Palermo was administered a questionnaire aimed at collecting their socio-demographic data and sexual behavior. In this retrospective study, we analyzed both the overall sample and four subgroups, created based on the sexual orientation of subjects, with the aims of finding associations between socio-demographic characteristics and risky behaviors and outlining a profile of patients attending our unit. These findings may provide contribution to STI epidemiology and improve prevention and care strategies in Italy.

## 2. Materials and Methods

Trained nurses administered a structured questionnaire to patients attending the STI Unit of the Policlinico “Paolo Giaccone” in Palermo (Sicily, Italy) from January 2018 to March 2019. All subjects were required to provide their informed consent before being included in the study. The study was conducted in accordance with the Declaration of Helsinki and approved by the Ethics Committee.

The variables collected were the following: socio-demographic data (age, sex, nationality, level of education, sexual orientation identity); clinical care received (the reason for the visit); risk indicators (age at first sexual intercourse, number of partners, use of the condom, sexual behavior, previous STIs, drug use, frequency of alcohol use, presence of comorbidities including HIV). Concerning sexual habits, the questionnaire asked patients whether they had had sex with male or female partner(s) in the last six months prior to the visit. At the analysis, the sample was then subdivided into four groups: MSM, MSW, bisexual men reporting sex with both males and females (bisexuals), and females (women reporting sex with men).

A preliminary interview and clinical examination wasconducted for all patients; in particular, demographics, medical history (co-infections, co-morbidities and antiretroviral treatments—ARTs), imaging and clinical data at the time of STI diagnosis were collected from existing medical records. After that, all patients were tested for chlamydia (*Chlamydia trachomatis*), syphilis (*Treponema pallidum*), *Mycoplasma genitalium* and *Trichomonas vaginalis* infections, genital herpes (Herpes Simplex Virus, HSV), HPV infection (human papillomavirus) and AIDS (Human Immunodeficiency Virus, HIV).

We carried out microbiological diagnostics following standard procedures established in STI screening protocols, including quality control measures.

*C. trachomatis* and *M. genitalium* were detected with real-time multiplex PCR using Anyplex™ II STI- 7 (Seegene Inc., Seoul, Korea), following the manufacturer’s instructions [18].

*T. pallidum* testing was conducted on all participants using a commercially available standard Rapid Plasma Reagin (RPR) test (Diagnostic International Distribution, IT), following the manufacturer’s instructions [19].

Human papillomavirus was detected by reverse hybridization (Linear ArrayHPV Genotyping Test, Roche Diagnostics), which allows the identification of 37 anogenital HPV types: 16 types are considered as lrHPV (low-risk HPV)(HPV 6, HPV 11, HPV 40, HPV 42, HPV 54, HPV 55, HPV 61, HPV 62, HPV 64, HPV 71, HPV 72, HPV 81, HPV 83, HPV 84, HPV 87 and HPV 89) and 21 types (HPV 16, HPV 18, HPV 26, HPV 31, HPV 33, HPV 35, HPV 39, HPV 45, HPV 51, HPV 52, HPV 53, HPV 56, HPV 58, HPV 59, HPV 66, HPV 67, HPV 68, HPV 69, HPV 70, HPV 73 and HPV 82) are considered as definitive or probable hrHPV (high-risk HPV types) [20,21].

Data were first gathered in a sheet specifically created and then transferred to a computerized database. For quantitative variables, descriptive statistics were computed (mean, median, standard deviation). The frequency and percentage of STIs diagnosed in the overall sample and in the four subgroups were calculated. We then determined whether there were significant differences in STI prevalence, socio-demographic and sexual behavior characteristic among the groups. Inferential statistics methods (Pearson’s χ2test and Fisher’s exact test) were used to analyze each population group’s variables according to their socio-demographic and sexual behavior characteristics. A *p*-value of ≤0.05 was considered significant. All analyses were performed using StatSoft software (Stat- Soft^®^, version 10; Dell, Tulsa, OK, USA).

## 3. Results

During the study period, we identified 294 patients with confirmed STI among the subjects attending the STI Unit.

The general characteristics of the overall sample and the four subgroups, stratified according to sexual orientation, are reported in Table 1 (mean ± sd, or percentages). The male/female ratio was about 2:1 (205 males and 89 females). MSM accounted for 34.3% (101/294), MSW for 29.6% (87/294), bisexual men for 5.8% (17/294) and females for 30.3% (89/294).

Mean age was higher for MSW (47.9 ± 16.06) compared to MSM (39.99± 10.87), bisexuals (38.52 ± 9.01) and females (32.36 ± 11.59). For females, the group mean age was lower compared to the other groups.

For MSW, there were relatively more attendees from the older age groups (44.8% for >50 years old), while for MSM more attendees were from the 30–50 age range (29.7% for the 30–39 age range and 30.7% for the 40–50 age range) as in the bisexual group (41.2% for both 30–39 and 40–50 age range); instead, women attendees were younger (48.3% for 18–29 years old). In addition, 79.6% of the total attendees were of Italian ethnicity: among MSM and MSW, Italian was the most represented group (93% and 96.5%, respectively), while women were 50.6% Italian and 44.9% African, and bisexual men were 64.7% Italian and 29.4% African.

A higher education level was recorded for 44.5% of the overall sample, in particular for 52.5% of MSM and 52.8% of MSW. The female group was, instead, more heterogeneous: 21.3% had no education, and 30.3% had attended primary/secondary school and high school.

The main reason for patients attending the clinic was the presence of symptoms (226/294, 76.9%), followed by having risky sexual behavior (multiple partners, no use of condom, group sex violence), in particular for the female group (37%).

The mean age at first intercourse was 15.24 ± 2.18 for the whole sample. The mean age was similar for MSW and women, but it was significantly lower for MSM and bisexual men (14.63 ± 1.84 and 13.58 ± 1.76, respectively).

The number of partners in the 6 months prior to the visit was 1 and 5–10 for 25.5% and 20.7% of the overall sample, respectively. In the MSM group, 23.8% and 22.8% of subjects stated having 5–10 and >10 partners, respectively, whereas 43.7% of MSW subjects reported having 1 partner. As for the female group, 1 partner was reported by 27% and 5–10 partnersby 24.7% of subjects.

The use of acondom was lowoverall. A total of 48.3% of patients reported not using condoms and 42.5% using it “sometimes”. Furthermore, 56.3% of MSW, 76.5% of bisexual men and 49.8% of females reported not having used condoms in the last 6 months, while 55.4% of MSM used condoms “sometimes”. Only 22.8% of the overall sample had used a condom at the last intercourse.

Across the sample, 12.9% had an STI in the 6 months prior tothe visit, particularly 19.8% of MSM and 35.3% of bisexual men. Regarding sexual behavior, vaginal sex was reported by 97.7% and 98.9% of MSW and women, respectively, while MSM subjects reported anal sex and oral sex in 99% and 70.3% of cases, respectively. Oral sex was also practiced by 55% of females and 76.5% of bisexual men.

A small percentage of patients used intravenous drugs, while 17% reported using other drugs, the higher rate belonging to MSM (22.7%) and to the bisexual group (35.3%).

The frequency of alcohol use in a month was 1–3 times for 43.2% of the overall sample. In the female group, 55% reported no use of alcohol.

Previous HIV infection, considered as a risk factor, was detected in 33% of patients and in a significantly higher percentage among MSM (58.4%) and bisexuals (47%) compared to other groups.

### Frequency of STIs

Diagnosed STIs were distributed as shown in Table 2: HPV infection and syphilis were the most common infections (55.1% and 45.6%, respectively). In particular, HPV was detected more frequently in females and MSW (64% and 58.6%, respectively), while syphilis was more common in MSM (69.3%). Multiple STIs were found in 6.4% of subjects, mainly among MSM (14.8%) and bisexuals (23.5%). The most frequently detected co-infection was HPV plus syphilis. *M. genitalium*, *C. trachomatis* and HSV were found at low rates in our overall sample (3.7%, 3.4% and 0.7%, respectively).

## 4. Discussion

STI care should be managed within the frame of primary health care, i.e., with a traditional approach including a spectrum of services (health promotion by general practitioners, nurses, pharmacists and telephone helpline support). For STI patients needing more specialized care from different professional figures (infectious disease clinicians, microbiologists, dermatologists, gynecologists, urologists, social service and psychological support), a coordination system is needed that acts as a network and ensures ease of access to care.

In Italy, in the last twenty years, we have witnessed the failure of the primary health care system, resulting in a failure to treat STI patients, including pregnant women, newborns and families, not to mention other categories of frail subjects, such as adolescents and sexually abused individuals. On these premises, we suggest implementation of the primary health care system and completion of risk assessments in order to identify those patients for whom sexual health or STI-related education may be indicated. All STD staff need to implement counselling topics and recommendations to share with patients, as well as tips on how to approach sexual health education/counselling, thereby promoting patient-centered approaches and educational programs.

Our data show that the profile of STI patients attending the hospital in Palermo was prevalently composed of adult Italian men with a high level of education. Patients visited the clinic because of the presence of STI symptoms in the first place and, secondly, due to risky sexual behaviors. Only 12.9% of subjects had a diagnosis of STI in the previous 6 months, and more than three-quarters of the sample reported no previous STI.

Considering behavioral risk factors, our patients had their first sexual intercourse at a young age (around 15 years) and this is not uncommon, as it has been reported from other similar studies conducted in Spain as well [22,23]. It is well known that early sexual debut promotes the occurrence of risky sexual behaviors, as well as an increased risk of contracting STIs [24,25].

In terms of sexual orientation, our patients predominantly consisted of MSM (34.3%), in line with an increase in this population that may be linked to progressive reduction of social discrimination, leading to the increased disclosure of sexual orientation [26].

Among risk indicators for STIs, condom usage and number of partners are the most useful and used. In our study, we analyzed the use of a condom during the six months preceding the visit and at the time of the last sex intercourse. Our data pointed to inconsistent and infrequent condom usage. Concerning the number of partners, evaluated in the same period, our data recorded two opposite trends: 25.5% of patients reported 1 partner and 20.7% between 5 and 10 partners. Thus, both risk factors (inconsistent condom usage and having a large number of sexual partners) were predictors of STI risk in our population. These trends were also described by other studies performed in other countries [27,28,29]. The said predictors of STI risk are particularly accentuated in the MSM and bisexual groups; in fact, these minorities are known to be at high risk [30,31,32]. This scenario is aggravated by the presence of HIV, detected in 33% of the overall population (58.4% of MSM and 47% of bisexuals), as well as by 97% of HIV-positives reporting not having used a condom during the last intercourse and 54.6% using condoms only “sometimes” (data not shown). This alarming evidence is in agreement with other studies. This behavior could also be attributed to those HIV-positive subjects on antiretroviral therapy who have undetectable viral load and erroneously think they cannot transmit the disease [33,34,35]. These data are in line with those of authors who reported a recent increase in MSM attending STI clinics and a rise in infection rates coinciding with the introduction of cART (combination antiretroviral therapy) [11,35,36].

Another interesting aspect concerns oral sex, with this practice being reported by most of the population in the study (regardless of the group). In the absence of appropriate barriers (condoms or dental dams), this practice is associated with a significant risk for STI transmission, although patients are not aware of this risk [37].

Drug and alcohol use, reported by other investigators as a risk factor due to the negative impact on protective behavior, did not significantly affect the results of our study [38,39].

Subgroup analysis allowed some interesting considerations, the most prominent of which being that the number of homosexual and bisexual men visiting the STI Unit together represented the majority of our sample (34.3% and 5.8%, respectively).

MSM is a sexual group that have a significantly higher risk of contracting STIs compared to the general population, and it is well documented that HIV infections occur mainly in this group both in Europe and the USA, as confirmed by our data [30,31,32]. Other studies suggest that the issue of STIs in the MSM population has been increasing in recent decades, mainly due to risky behaviors [11,35,36]. Authors reported that MSM develop risky sexual behaviors [30,31,32], and this was confirmed in our study. MSM and bisexual subgroups included the highest percentage of subjects with HIV, a tendency to have numerous partners, occasional use of condom and, consequently, the highest frequency of STI diagnosis in the six months before the visit.

Other interesting considerations can be made regarding the female group. Almost half of the subjects in this group were younger compared to the other groups and were African. Furthermore, their level of education was lower compared to the other groups. Regarding the number of partners in the 6 months prior to the visit, the female group showed a trend similar to that of the MSM group, with 27% of subjects reporting 1 partner and 24.7% reporting 5 to 10 partners. Among females with multiple partners, 20/22 were African.

STIs are one of the leading public health problems affecting migrant women. Considering the spread of migration in Southern Italy and the risky sexual behaviors reported from our female group, it could be speculated that the African women in our sample were migrants and/or female sex workers [40]. Despite public health care being available to all migrants in Italy, very few data are available regarding STI surveillance among migrant women, especially paperless women. Moreover, no national preventive action targeted at the sexual health of female migrants has been implemented yet [31,32,33,34,35,36,37,38,39,40,41,42,43,44].

HPV infection and syphilis were the most prevalent STIs diagnosed in our unit. Co-infection was found in 5.8% of the overall sample, and in 12.9% and 17.6% of MSM and bisexual men, respectively. In general, HPV infections represent the most reported STI in Italy; the cases increased by two and a half times between 2004 and 2013, while the trend has been stabilizing since 2013 [4]. Our study revealed a very high prevalence of HPV across all groups, especially in the female one, and these data were higher than those recorded in other Italian reports [4]. Such a finding is probably due to sociocultural factors, as increasing immigration rates, rising sexual promiscuity and an increase in immigrant sex workers [4,43].

Syphilis was the second highest STI in terms of prevalence in our sample. In agreement with recent reports, we found that confirmed syphilis cases were detected, for the most part, in MSM (69.3%) [45,46,47]. Syphilis rates reported in Europe in 2016 were eighttimes higher in men than in women, and more than 60% of syphilis cases worldwide are found in the MSM population, as confirmed byour data [47,48,49].

The major limitation of this study consisted in enrolling patients who hadbeen referred to hospital services such as ambulatory care practices for HIV infections. As such, all STI patients from primary health service houses and psychiatric services were excluded from the study.

## 5. Conclusions

Our analysis can help identify groups at risk for STIs and contribute to enriching the limited and fragmented Italian data relating to STI prevalence and risk factors. The continuous increase in the rate of new cases of syphilis, specifically among MSM, reaffirms the need for screening programs targeted to this high-risk group.

Implementing the primary health care system and completing risk assessments could help identify those patients for whom sexual health or STI-related education may be indicated. All STD teams need to implement counselling topics and recommendations to share with patients, as well as tips on how to approach sexual health education/counselling, thereby promoting patient-centered approaches and educational programs.

## Figures and Tables

**Table 1:** Sociodemographic and behavioral characteristics of the sample.

Variable	Total	MSM	MSW	Bisexuals	Females	*p*-Value
	(*n* = 294)	(*n* = 101, 34.3%)	(*n* = 87, 29.6%)	(*n* = 17, 5.8%)	(*n* = 89, 30.3%)	
Age:						
mean ± sd	49.9 ± 14.05	39.9 ± 10.87	47.9 ± 16.06	38.5 ± 9.01	32.36 ± 1.59	N.A.
Age range: *n* (%)						
18–29	79 (26.9)	20 (19.8)	14 (16.1)	2 (11.8)	43 (48.3)	N.A.
30–39	79 (26.9)	30 (29.7)	16 (18.9)	7 (41.2)	26 (29.2)	N.A.
40–50	69 (23.5)	31 (30.7)	18 (20.7)	7 (41.2)	13 (14.6)	N.A.
>50	67 (22.8)	20 (19.8)	39 (44.8)	1 (5.9)	7 (7.9)	N.A.
Race/Ethnicity: *n* (%)						
Italy	234 (79.6)	94 (93.0)	84 (96.5)	11 (64.7)	45 (50.5)	<0.01
Africa	51 (17.3)	3 (2.9)	3 (3.4)	5 (29.4)	40 (44.9)	<0.01
Other	9 (3.0)	4 (3.9)	0	1 (5.9)	4 (4.5)	0.555
Education: *n* (%)						
No education	30 (10.2)	5 (4.9)	2 (2.3)	4 (23.5)	19 (21.3)	<0.01
Primary/Secondary	65 (22.1)	14 (13.9)	20 (22.9)	4 (23.5)	27 (30.3)	0.056
High	131 (44.5)	53 (52.5)	46 (52.8)	5 (29.4)	27 (30.3)	<0.01
Tertiary	68 (23.1)	29 (28.7)	19 (21.8)	4 (23.5)	16 (17.9)	0.364
Reasonof examination						
Symptoms	226 (76.9)	68 (67.3)	75 (86.2)	11 (64.7)	72 (80.9)	<0.01
Partner with STI	11 (3.7)	5 (4.9)	3 (3.4)	1 (5.8)	2 (2.24)	0.753
Risky sexual behavior	80 (27.2)	28 (27.7)	13 (14.9)	6 (35.2)	33 (37.0)	<0.01
Other	34 (11.5)	18 (17.8)	5 (5.7)	3 (17.6)	8 (8.9)	0.047
Behavioral risk factors						
Age at first intercourse:						
mean ± sd	15.24 ± 2.18	14.63 ± 1.84	15.88 ± 1.93	13.58 ± 1.76	15.07 ± 2.01	<0.01
No. of sex partners						
(last 6 months)						
0	4 (1.3)	0	4 (4.59)	0	0	N.A.
1	75 (25.5)	12 (11.8)	38 (43.6)	1 (5.9)	24 (26.9)	<0.01
2	35 (11.9)	8 (7.92)	14 (16.1)	1 (5.9)	12 (13.4)	0.285
3	44 (14.9)	16 (15.8)	13 (14.9)	1 (5.9)	14 (15.7)	0.752
4	40 (13.6)	18 (17.8)	7 (8.0)	2 (11.7)	13 (14.6)	0.268
5–10	61 (20.7)	24 (23.8)	10 (11.49)	5 (29.4)	22 (24.7)	0.081
>10	35 (11.9)	23 (22.8)	1 (1.1)	7 (41.1)	4 (4.5)	<0.01
Condom use						
(last 6 months)						
Usually	26 (8.8)	9 (8.9)	12 (13.8)	2 (1.7)	3 (3.3)	0.105
Sometimes	125 (42.5)	56 (55.4)	26 (29.9)	3 (17.6)	40 (44.9)	<0.01
No	142 (48.3)	36 (35.6)	49 (56.3)	13 (76.5)	44 (49.8)	<0.01
At last sexual intercourse	67 (22.8)	25 (24.7)	21 (24.1)	4 (23.5)	17 (19.0)	0.80
Sexual behavior						
Vaginal sex	189 (64.3)	0	85 (97.7)	12 (70.6)	88 (98.9)	N.A.
Oral sex	166 (56.4)	71 (70.3)	9 (10.3)	13 (76.4)	49 (55.0)	<0.01
Anal sex	156 (53.0)	100 (99)	33 (37.9)	15 (29.4)	32 (35.9)	<0.01
STIs in the last 6 months	38 (12.9)	20 (19.8)	8 (9.2)	6 (35.3)	4 (4.5)	<0.01
Drug use						
Intravenous	6 (2.0)	3 (2.9)	1 (1.14)	1 (5.88)	1 (1.12)	0.491
Other	50 (17.0)	23 (22.7)	10 (11.5)	6 (35.3)	11 (12.4)	0.021
Frequency of alcohol use						
(in one month)						
0	89 (30.2)	9 (8.91)	30 (34.5)	1 (5.8)	49 (55.0)	<0.01
1–3	127 (43.2)	45 (44.5)	42 (48.2)	9 (52.9)	31 (34.8)	0.242
4–7	50 (17.0)	29 (28.7)	11 (12.6)	1 (5.8)	9 (10.1)	<0.01
9–20	18 (6.1)	14 (13.8)	3 (3.4)	1 (5.8)	0	N.A.
>20	10 (3.4)	4 (3.9)	1 (1.1)	5 (29.4)	0	N.A.
Comorbidities						
HIV	97 (33)	59 (58.4)	20 (22.9)	8 (47.0)	10 (11.2)	<0.01
HBV	4 (1.3)	2 (1.9)	0	1 (5.8)	1 (1.1)	N.A.
HCV	5 (1.7)	3 (2.9)	2 (2.3)	0	0	N.A.
Other	12 (4.0)	12 (11.8)	7 (8.0)	3 (17.6)	2 (2.2)	0.044
None	184 (62.5)	43 (42.5)	60 (68.9)	7 (41.1)	76 (85.4)	<0.01

**Table 2:** Prevalence of sexually transmitted infections (STIs).

STIs	Total	MSM	MSW	Bisexuals	Females	*p*-Value
*n* (%)	(*n* = 294)	(*n* = 101)	(*n* = 87)	(*n* = 17)	(*n* = 89)	
HPV	162 (55.1)	45 (44.5)	51 (58.6)	9 (52.9)	57 (64.0)	0.048
Syphilis	134 (45.6)	70 (69.3)	28 (32.1)	11 (64.7)	25 (28.0)	<0.01
Stage:						
primary	25 (8.5)	16 (15.8)	2 (2.3)	2 (11.7)	5 (5.6)	<0.01
secondary	67 (22.7)	31 (30.7)	17 (19.5)	6 (35.3)	13 (14.6)	0.029
latent	45 (15.3)	26 (25.7)	9 (10.3)	3 (17.6)	7 (7.8)	<0.01
*M. genitalium*	11 (3.7)	1 (0.9)	3 (3.4)	0	7 (7.8)	N.A.
*C. trachomatis*	10 (3.4)	1 (0.9)	5 (5.7)	0	4 (4.4)	N.A.
*HSV*	2 (0.7)	1 (0.9)	0	1 (5.88)	0	N.A.
Other	3 (1.0)	1 (0.9)	0	0	1 (1.1)	N.A.
Multiple STIs *	19 (6.4)	15 (14.8)	1 (1.14)	4 (23.5)	3 (3.3)	<0.01
HPV + Syphilis	17 (5.7)	13 (12.8) ^C^	1 (1.14) ^C^	3 (17.6) ^C^	0	<0.01 ^C^
HPV + *C. trachomatis*	1 (0.3)	0	0	0	1 (1.1)	N.A.
HPV + HSV (genital herpes)	1 (0.3)	0	0	1 (5.8)	0	N.A.
HPV + *C. trachomatis* + *M. genitalium*	2 (0.6)	0	0	0	2 (2.2)	N.A.
HPV + Syphilis + *M. genitalium*	1 (0.3)	1 (0.9)	0	0	0	N.A.
HPV + Syphilis + HSV (genital herpes)	1 (0.3)	1 (0.9)	0	0	0	N.A.

* Multiple STIs: having two or more of the screened STIs. ^C^ MSM vs. MSW, MSM vs. Bisexual, MSW vs. Bisexual.

## Data Availability

The data used to support the findings of this study are available from the corresponding author upon request.
